# Cost-Effectiveness of First-Line Immunochemotherapy Versus *BRAF* Plus *MEK* Inhibitors in *BRAF*^V600E^-Mutated Metastatic Lung Cancer

**DOI:** 10.3390/curroncol33070384

**Published:** 2026-06-24

**Authors:** Chian-Wei Chen, Jui-Hung Tsai, Sheng-Han Tsai, Li-Jun Chen, Szu-Chun Yang

**Affiliations:** 1 Department of Internal Medicine, National Cheng Kung University Hospital, College of Medicine, National Cheng Kung University, Tainan 704, Taiwan; 2 Department of Oncology, National Cheng Kung University Hospital, College of Medicine, National Cheng Kung University, Tainan 704, Taiwan; 3 National Cheng Kung University Hospital Cancer Center, College of Medicine, National Cheng Kung University, Tainan 704, Taiwan; 4 Department of Statistics, College of Management, National Cheng Kung University, Tainan 701, Taiwan

**Keywords:** *BRAF* mutation, cost-effectiveness, lung cancer, dabrafenib, trametinib, immunotherapy

## Abstract

Patients with *BRAF*^V600E^-mutated metastatic lung cancer benefit from both *BRAF* plus *MEK* inhibitors and immune checkpoint inhibitor (ICI)–chemotherapy. This study evaluated the cost-effectiveness of first-line ICI–chemotherapy compared with *BRAF* plus *MEK* inhibitors in these patients. This economic analysis, with a 15-year time horizon and an annual 3% discount, was conducted from the perspective of the healthcare sectors in Taiwan and the US. Simulated patients were entered into partitioned survival models upon initiation of first-line therapies. The model inputs were derived from the FRONT-BRAF study (progression-free/overall survival, adverse events, and subsequent therapies), insurance payments or retail prices (costs of drugs, physician visits, monitoring, adverse events, and end-of-life care), and a hospital cohort (health utility). Deterministic and probabilistic analyses were performed. The incremental cost-effectiveness ratios (ICERs) of ICI–chemotherapy compared with *BRAF* plus *MEK* inhibitors (Taiwan: $73,561/QALY; US: $290,279/QALY) exceeded the willingness-to-pay (WTP) thresholds (Taiwan: $70,000/QALY; US: $150,000/QALY). The drug costs of subsequent therapies and the utility values of the progressive-disease state were the major determinants of ICERs. In Taiwan, ICI–chemotherapy had a 41.0% probability of being cost-effective at the WTP threshold. ICI–chemotherapy had a higher probability of being cost-effective than *BRAF* plus *MEK* inhibitors when the WTP exceeded $300,000/QALY in the US. Our analysis suggests that, despite the longer survival of first-line ICI–chemotherapy compared with *BRAF* plus *MEK* inhibitors, ICI–chemotherapy is not a cost-effective strategy for patients with *BRAF*^V600E^-mutated metastatic lung cancer.

## Simple Summary

Patients with *BRAF*^V600E^-mutated metastatic lung cancer benefit from first-line *BRAF* plus *MEK* inhibitors and immune checkpoint inhibitor–chemotherapy. Nevertheless, a cost-effectiveness analysis comparing both treatments is lacking. This study suggests that despite the longer survival of first-line immune checkpoint inhibitor–chemotherapy compared with *BRAF* plus *MEK* inhibitors, the incremental cost-effectiveness ratios (Taiwan: $73,561/QALY; US: $290,279/QALY) exceeded the respective willingness-to-pay thresholds. Namely, first-line immune checkpoint inhibitor–chemotherapy is not a cost-effective strategy for patients with *BRAF*^V600E^-mutated metastatic lung cancer.

## 1. Introduction

The v-Raf murine sarcoma viral oncogene homolog B, Val600Glu (*BRAF*^V600E^) mutation is found in 1–3% of non-small cell lung cancer (NSCLC) [1]. Patients with *BRAF*^V600E^-mutated metastatic NSCLC benefit from *BRAF* plus mitogen-activated protein kinase (*MEK*) inhibitors [2]. Dabrafenib plus trametinib were found to have a durable effect in treatment-naïve and previously treated patients [3,4,5], whereas encorafenib plus binimetinib showed even longer overall survival (OS) in untreated patients [6,7]. Additionally, *BRAF*^V600E^ mutation is frequently associated with elevated programmed death-ligand 1 (PD-L1) expression levels, which makes tumors susceptible to immune checkpoint inhibitors (ICIs) [8].

To date, no head-to-head trials have compared first-line *BRAF* plus *MEK* inhibitors with ICI-based therapies in patients with *BRAF*^V600E^-mutated metastatic NSCLC. A German real-world study found that time to treatment failure and OS were identical for patients receiving first-line therapies with dabrafenib-trametinib and ICI alone/ICI–chemotherapy, and there was a trend toward superior median OS in patients receiving ICI–chemotherapy (ICI alone: 21.0 months, dabrafenib-trametinib: 28.0 months, and ICI–chemotherapy: 31.8 months) [9]. Another retrospective study across 17 centers and four countries found that ICI-based therapies were associated with identical progression-free survival (PFS, *p* = 0.69) but improved median OS (40.9 months) compared with *BRAF* plus *MEK* inhibitors (22.7 months, hazard ratio = 0.63, *p* = 0.04) in the propensity score-matched cohort [10]. These findings are of clinical relevance as a randomized controlled trial comparing outcomes of both treatment strategies might not be easily performed, given that *BRAF*^V600E^ is a relatively rare gene alteration in lung cancer.

Given that the economic burden of lung cancer has increased substantially over the past decade [11], selecting a cost-effective first-line treatment for patients with *BRAF*^V600E^-mutated metastatic NSCLC is important. Although first-line ICI–chemotherapy is associated with improved post-progression survival, subsequent therapy imposes substantial costs on the healthcare sector. Associated adverse events (AEs) also have financial consequences. Further investigation is required to determine whether the benefits outweigh the costs. To the best of our knowledge, there has been no economic analysis examining the cost-effectiveness of first-line ICI–chemotherapy compared with *BRAF* plus *MEK* inhibitors. Based on the results of the FRONT-BRAF study [10], we aimed to evaluate the cost-effectiveness of ICI–chemotherapy and *BRAF* plus *MEK* inhibitors as first-line treatments in patients with *BRAF*^V600E^-mutated metastatic lung cancer.

## 2. Materials and Methods

### 2.1. Model Overview

Partitioned survival models were adopted to simulate treatment-naïve patients with *BRAF*^V600E^-mutated metastatic lung cancer who received ICI–chemotherapy or *BRAF* plus *MEK* inhibitors as the first-line therapy (Supplementary Figure S1). We selected a model length of three weeks because ICI and chemotherapy are administered every three weeks. The time horizon was 15 years, and we applied an annual rate of 3% to discount future costs and life-years. This cost-effectiveness analysis followed the guidelines of the Consolidated Health Economic Evaluation Reporting Standards (CHEERS) (Supplementary Table S1) [12].

We selected pembrolizumab, pemetrexed, and carboplatin as the ICI–chemotherapy regimen and considered maintenance therapy with pembrolizumab and pemetrexed after four cycles of carboplatin. Based on the FRONT-BRAF study, 87.2% of patients received dabrafenib and trametinib, and 12.8% of patients received encorafenib and binimetinib as the *BRAF* plus *MEK* inhibitors [10]. After disease progression, 80.3% of patients starting with ICI–chemotherapy received subsequent therapy (24.5% chemotherapy, 75.5% *BRAF* and *MEK* inhibitors), whereas a smaller proportion (56.6%) of patients on *BRAF* and *MEK* inhibitors received subsequent therapy (13.1% chemotherapy, 84.4% ICI–chemotherapy, and 2.4% *BRAF* and *MEK* inhibitors) (Table 1).

### 2.2. Survival Estimates

We used web-based software (WebPlotDigitizer, version 5.2) [17] to extract PFS and OS curves for first-line ICI–chemotherapy and *BRAF* and *MEK* inhibitors in the propensity score-matched cohort of the FRONT-BRAF study. The PFS and OS data were translated into patient-level information using the function “*digitise()*” in *R* [18,19]. We fitted parametric (loglogistic, Weibull, lognormal, gamma, generalized gamma, exponential) models to the data and selected the one with the most appropriate fit based on the Akaike Information Criterion (AIC) or Bayes Information Criterion (BIC) to extrapolate PFS and OS beyond the study follow-up period. Accordingly, the proportions of patients with progression-free disease, progressive disease, or deaths in each cycle were derived. We compared the modeled PFS and OS curves of first-line ICI–chemotherapy and *BRAF* and *MEK* inhibitors with those of the FRONT-BRAF study [10].

### 2.3. Cost and Health Utility

This economic analysis was conducted from the perspective of the healthcare sectors in Taiwan and the US. The Taiwan National Health Insurance (NHI) program is a single-payer system in which the drug prices for immunotherapy and targeted therapy are low. However, limited resources are available for drug reimbursements. Most individuals in the US receive insurance from private companies. Drug prices are high. We considered direct medical costs for drugs, physician visits, general monitoring (hemogram, biochemistry tests, and chest radiography), computed tomography with contrast, and end-of-life care [13,14,15]. We used the example of a 65-year-old man with a serum creatinine level of 1.0 mg/mL, average body surface area of 1.68 (1.90) m^2^, body weight of 60 (90) kg, and glomerular filtration rate of 73 (84) mL/min, to estimate the doses of intravenous agents in Taiwan (the US). Drug waste was accounted for by rounding up partially used vials while calculating drug costs (Supplementary Table S2). The incidence rates of AEs for ICI–chemotherapy and *BRAF* and *MEK* inhibitors were directly derived from the FRONT-BRAF study [10]. We multiplied the incidence rates of AE by the costs of AE management to estimate the costs attributable to AEs under each therapy (Supplementary Table S3) [20,21]. All costs were made equivalent to 2025 US dollars (USD).

Based on a hospital cohort’s European Quality of Life-Five Dimensions (EQ-5D) data, we applied a health utility value of 0.80 for patients in the progression-free state under ICI–chemotherapy [16]. For patients in the progression-free state under *BRAF* and *MEK* targeted therapy, we assigned a health utility value of 0.84. Patients in the progressive disease state receiving ICI–chemotherapy or targeted therapy received a health utility value of 0.72. These values echo the health utilities in metastatic NSCLC patients under ICI–chemotherapy [22] and targeted therapy [23].

### 2.4. Base-Case Analysis

We estimated the incremental cost-effectiveness ratio (ICER) as the incremental cost divided by quality-adjusted life years (QALY). In accordance with previous Taiwanese studies [24,25,26,27], we selected a willingness-to-pay (WTP) threshold of 70,000 USD per QALY, which is approximately two per capita gross domestic products (GDPs) in 2025 [28] and consistent with the most prominent practice of 1 to 3 times the GDP per capita [29]. For the US, a WTP threshold of 150,000 USD per QALY was selected [30]. We calculated the net monetary benefit (NMB) of each strategy as the product of QALYs and WTP minus the total cost. The incremental NMB was defined as the difference in NMB between the two strategies.

### 2.5. Deterministic and Probabilistic Analyses

One-way deterministic analyses were performed by varying the model parameters within plausible ranges (Table 1 and Supplementary Table S3) to generate tornado diagrams. To address the effects of parameter uncertainties, probabilistic analyses using a Monte Carlo simulation with 1000 iterations were conducted to generate the acceptability curves. *R* version 4.5.1 (*R* Foundation, Boston, MA, USA) was used to perform all the analyses.

### 2.6. Exploratory Analysis of TP53 Mutant and Wild-Type Subgroups

The FRON-BRAF study demonstrated PFS and OS curves of subgroups with and without *TP53* co-mutations [10]. First-line ICI–chemotherapy, compared with *BRAF* plus *MEK* inhibitors, was associated with longer OS in patients with *TP53* co-mutations. However, first-line ICI–chemotherapy was associated with a shorter PFS in patients with wild-type *TP53*. We fitted the PFS and OS data of patients with *TP53* co-mutations and wild-type *TP53* for the exploratory analysis.

### 2.7. Sensitivity Analysis Using Alternative Models for Survival Extrapolation

The most common parametric models for OS extrapolation included Weibull, loglogistic, lognormal, and exponential models [31]. To test the validity of our results, we used these alternative models to extrapolate the OS for sensitivity analysis.

## 3. Results

### 3.1. Base-Case Analysis

Based on the AIC and BIC of each parametric model (Supplementary Table S4), we selected log-normal distributions to extrapolate the PFS of patients receiving first-line ICI–chemotherapy and *BRAF* plus *MEK* inhibitors. Log-normal and log-logistic distributions were selected to extrapolate the OS of patients undergoing first-line ICI–chemotherapy and *BRAF* plus *MEK* inhibitors, respectively. The modeled PFS and OS curves resembled those demonstrated in the FRONT-BRAF study [10], indicating a good fit for the selected parametric models (Supplementary Figure S2).

Patients receiving first-line ICI–chemotherapy incurred an additional 101,183 USD (US: 399,278 USD) and effected an additional 1.37 QALYs compared with those receiving *BRAF* plus *MEK* inhibitors (Table 2), resulting in an ICER of 73,561 USD per QALY (US: 290,279 USD per QALY). Drug costs constituted the main component of the total costs and the cost difference between the two strategies. Pre-progression (ICI–chemotherapy: 1.36, *BRAF* plus *MEK* inhibitors: 1.30) QALYs did not differ significantly; however, patients receiving first-line ICI–chemotherapy had much longer post-progression QALYs compared with those receiving *BRAF* plus *MEK* inhibitors (2.35 versus 1.04 QALYs). The incremental NMB of ICI–chemotherapy compared with *BRAF* plus *MEK* inhibitors was −4899 USD (US: −192,953 USD).

### 3.2. Deterministic and Probabilistic Analyses

**Figure 1:**
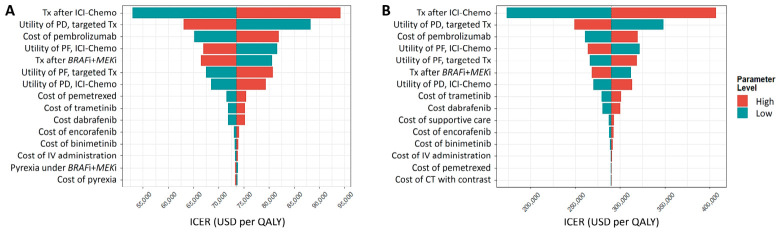
Tornado diagrams of ICI–chemotherapy versus *BRAF* plus *MEK* inhibitors ((**A**): Taiwan; (**B**): US). The dotted lines represent the base-case incremental cost-effectiveness ratios (ICERs). *BRAF*i, v-Raf murine sarcoma viral oncogene homolog B inhibitor; CT, computed tomography; ICI-Chemo, immune checkpoint inhibitor plus chemotherapy; IV, intravenous; *MEK*i, mitogen-activated protein kinase inhibitor; PD, progressive disease; PF, progression-free; QALY, quality-adjusted life-year; Tx, therapy; USD, US dollars.

Tornado diagrams of first-line ICI–chemotherapy compared with *BRAF* plus *MEK* inhibitors showed that the probability of subsequent therapy for the ICI–chemotherapy group was the major determinant of ICERs (Figure 1). Notably, given that less than 77.2% of patients receive subsequent therapy after disease progression, ICI–chemotherapy could become a cost-effective strategy in Taiwan. Additionally, the higher the health utility of the progressive-disease state under targeted therapy or the progressive-free state under ICI–chemotherapy, the lower the ICER values.

**Figure 2:**
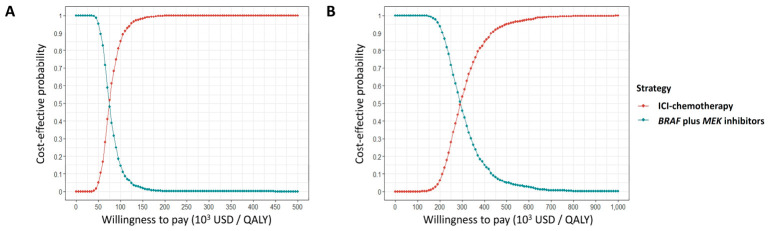
Acceptability curves for ICI–chemotherapy and *BRAF* plus *MEK* inhibitors ((**A**): Taiwan; (**B**): US). *BRAF*, v-Raf murine sarcoma viral oncogene homolog B; ICI, immune checkpoint inhibitor; *MEK*, mitogen-activated protein kinase; QALY, quality-adjusted life-year; USD, US dollars.

In Taiwan, the ICI–chemotherapy strategy had 41.0% and 89.0% probabilities of being cost-effective at the WTP threshold of 70,000 USD (2 per capita GDP) and 105,000 USD (3 per capita GDP) per QALY, respectively (Figure 2). In the US, first-line ICI–chemotherapy was unlikely to be cost-effective at the WTP threshold of 150,000 USD per QALY. If the WTP threshold was increased to 300,000 USD per QALY, ICI–chemotherapy had a higher probability of being cost-effective compared with *BRAF* plus *MEK* inhibitors.

### 3.3. Exploratory Analysis of TP53 Mutant and Wild-Type Subgroups

In the *TP53* mutant subgroup, patients receiving first-line ICI–chemotherapy had longer (3.96 − 1.95 = 2.01) QALYs than those receiving *BRAF* and *MEK* inhibitors. The ICER was 70,528 USD per QALY (US: 307,133 USD per QALY), whereas the incremental NMB was −960 USD (US: −315,846 USD, Table 3). However, in the *TP53* wild-type subgroup, patients receiving first-line *BRAF* and *MEK* inhibitors had slightly longer QALYs than those receiving ICI–chemotherapy. The ICER of *BRAF* and *MEK* inhibitors compared with ICI–chemotherapy was 90,924 USD per QALY (US: 696,767 USD per QALY), with an incremental NMB of −9356 USD (US: −244,486 USD).

### 3.4. Sensitivity Analysis Using Alternative Models for Survival Extrapolation

Varying the models to extrapolate the OS of patients receiving ICI–chemotherapy or *BRAF* plus *MEK* inhibitors, the ICERs were similar to those in the base case (Supplementary Table S5).

## 4. Discussion

The selection of ICI–chemotherapy or *BRAF* plus *MEK* inhibitors as first-line therapy for patients with *BRAF*^V600E^-mutated metastatic lung cancer is an issue under constant debate. Although real-world studies have found that first-line ICI–chemotherapy improved OS compared with *BRAF* plus *MEK* inhibitors [9,10], cost-effectiveness analyses comparing both treatment strategies have not been conducted. Based on the FRONT-BRAF study [10], our economic analysis demonstrated that first-line ICI–chemotherapy greatly increased the post-progression QALYs and drug costs of subsequent therapy. The ICERs of first-line ICI–chemotherapy compared with *BRAF* and *MEK* inhibitors exceeded the WTP thresholds in both countries (Taiwan: 73,561 USD per QALY; US: 290,279 USD per QALY). Probabilistic analysis and sensitivity analysis using alternative models to extrapolate the OS further validated that first-line ICI–chemotherapy is unlikely to be cost-effective.

In line with two important cohort studies [9,10], the pre-progression life years of ICI–chemotherapy and *BRAF* plus *MEK* inhibitors were similar. Longer post-progression life years of the ICI–chemotherapy group might reflect the delayed and sustainable benefits commonly observed in ICI-based therapies [32]. Owing to a major life-year difference in the post-progression state, subsequent drug costs and utility values of the progressive-disease state play important roles in determining the cost-effectiveness. For example, if the ICI–chemotherapy (*BRAF* plus *MEK* inhibitors) group’s probability of subsequent therapy could be decreased (increased) to 77.2% (62.4%) without affecting the effectiveness, first-line ICI–chemotherapy would become a cost-effective strategy in Taiwan. Similarly, given a utility value of the progressive-disease state under targeted therapy of >0.74, the ICER could be lower than the WTP threshold.

Probabilistic analyses of the two different healthcare environments revealed that first-line ICI–chemotherapy was unlikely to be cost-effective under the respective WTP thresholds. Given the increase in healthcare spending on cancer care, we acknowledge that the WTP thresholds might be underestimated. Researchers have inferred higher WTP thresholds, such as 3 per capita GDP per QALY in Taiwan and 300,000 USD per QALY in the US [33,34]. Our results indicated that first-line ICI–chemotherapy had an 89.0% probability of being cost-effective at the elevated WTP threshold in Taiwan; however, it only had a 54.0% probability of being cost-effective at the WTP threshold of 300,000 USD in the US. Such a discrepancy might result from the lower drug cost of subsequent therapy in Taiwan compared to that in the US, reducing the incremental cost and enhancing cost-effectiveness in Taiwan.

Exploratory analysis of *TP53* wild-type patients showed that first-line ICI–chemotherapy no longer resulted in longer QALYs than *BRAF* plus *MEK* inhibitors. Patients without *TP53* co-mutations may be less likely to develop resistance to *BRAF* plus *MEK* inhibitors and have a better OS than those with *TP53* co-mutations. The OS benefit of first-line ICI–chemotherapy was mainly driven by the *TP53* mutant subgroup. Nevertheless, the ICERs still exceeded the WTP thresholds. As the PFS of ICI–chemotherapy and *BRAF* plus *MEK* inhibitors were similar in the *TP53* mutant subgroup [10]; a plausible reason for the high ICERs was the high cost incurred for subsequent therapy.

This study has several limitations. First, the PFS and OS were not simulated from a randomized controlled trial comparing first-line ICI–chemotherapy with *BRAF* plus *MEK* inhibitors. Although we used PFS and OS data in the propensity score-matched cohort of the FRONT-BRAF study [10], residual confounding still existed after balancing the age, smoking status, Eastern Cooperative Oncology Group performance status, PD-L1 expression levels, and brain metastasis of the two treatment groups. Patients receiving first-line ICI–chemotherapy were more likely to be healthier than those receiving *BRAF* plus *MEK* inhibitors. As a result, the survival benefit of ICI–chemotherapy might be overestimated, leading to a lower ICER value. Second, we did not consider dose reduction or interruption of therapy, leading to an overestimation of drug costs. This phenomenon is particularly apparent in subsequent ICI–chemotherapy after first-line *BRAF* plus *MEK* inhibitors, as more AEs leading to dose reduction or interruption are thought to occur in this state. Dose reduction or interruption would lower the costs for patients receiving first-line *BRAF* plus *MEK* inhibitors, thus making first-line ICI–chemotherapy less likely to be cost-effective. Third, exploratory analysis according to *TP53* mutation status was based on small retrospective subgroups without propensity score matching; the results should be interpreted cautiously.

## 5. Conclusions

In conclusion, despite the longer OS of first-line ICI–chemotherapy compared with *BRAF* plus *MEK* inhibitors in a real-world scenario [10], our analysis suggests that first-line ICI–chemotherapy is unlikely to be a cost-effective strategy for patients with *BRAF*^V600E^-mutated metastatic lung cancer. Selecting subsequent therapies to reduce drug costs and increase health utility in the progressive-disease state would enhance cost-effectiveness.

## Figures and Tables

**Table 1:** Model parameters.

Parameters	Value	Range	Distribution	Source
Proportion of patients in each state				
ICI–chemotherapy	time-variant	--	--	FRONT-BRAF study [10]
*BRAF* plus *MEK* inhibitors	time-variant	--	--	FRONT-BRAF study [10]
Subsequent therapy				
ICI–chemotherapy:	80.3%	64.2–96.4%	beta	FRONT-BRAF study [10]
Chemo, targeted therapy	24.5, 75.5%	--	Dirichlet	FRONT-BRAF study [10]
*BRAF* plus *MEK* inhibitors:	56.6%	45.3–67.9%	beta	FRONT-BRAF study [10]
Chemo, ICI-Chemo, targeted therapy	13.1, 84.4, 2.4%	--	Dirichlet	FRONT-BRAF study [10]
Taiwan-Unit costs, USD				
IV drug administration	74	59–89	gamma	NHI payment
Drug cost per 3 weeks:				
Carboplatin	160	128–192	gamma	NHI payment
Pembrolizumab	3289	2631–3947	gamma	NHI payment
Pemetrexed	500	400–600	gamma	NHI payment
Dabrafenib	1781	1425–2137	gamma	NHI payment
Trametinib	1782	1425–2138	gamma	NHI payment
Encorafenib	3578	2862–4294	gamma	NHI payment
Binimetinib	2734	2187–3281	gamma	Retail price
Physician visit	10	8–11	gamma	NHI payment
General monitoring per 3 weeks	22	18–27	gamma	NHI payment
CT with contrast	168	134–202	gamma	NHI payment
End-of-life care	3228	2583–3874	gamma	NHI payment
US-Unit costs, USD				
IV drug administration	196	157–235	gamma	CMS, CPT96413 and CPT96415 [13]
Drug cost per 3 weeks:				
Carboplatin	53	42–64	gamma	Drugs.com [14]
Pembrolizumab	11,538	9230–13,845	gamma	Drugs.com [14]
Pemetrexed	99	79–119	gamma	Drugs.com [14]
Dabrafenib	10,763	8610–12,915	gamma	Drugs.com [14]
Trametinib	11,685	9348–14,022	gamma	Drugs.com [14]
Encorafenib	16,813	13,450–20,176	gamma	Drugs.com [14]
Binimetinib	10,998	8798–13,198	gamma	Drugs.com [14]
Supportive care per 3 weeks	602	482–722	gamma	SEER-Medicare analysis [15]
CT with contrast	193	154–232	gamma	CMS, CPT71270 [13]
End-of-life care	11,886	9508–14,263	gamma	SEER-Medicare analysis [15]
Health utility				
Progression free, ICI-Chemo	0.80	0.72–0.88	beta	EQ-5D [16]
Progression free, targeted therapy	0.84	0.76–0.92	beta	EQ-5D [16]
Progressive disease, ICI-Chemo	0.72	0.65–0.79	beta	EQ-5D [16]
Progressive disease, targeted therapy	0.72	0.65–0.79	beta	EQ-5D [16]

*BRAF*, v-Raf murine sarcoma viral oncogene homolog B; CMS, Centers for Medicare & Medicaid Services; CPT, Current Procedure Terminology; CT, computed tomography; EQ-5D, European Quality of Life-Five Dimensions; ICI-Chemo, immune checkpoint inhibitor plus chemotherapy; IV, intravenous; *MEK*, mitogen-activated protein kinase; NHI, National Health Insurance; SEER, Surveillance, Epidemiology, and End Results Program; USD, US dollars.

**Table 2:** Base-case results.

Strategy	Costs (USD)		Life Years	QALYs	Incremental Cost per Life Year (USD)		Incremental Cost per QALY (USD)		INMB (USD)	
	Taiwan	US			Taiwan	US	Taiwan	US	Taiwan	US
*BRAF* plus *MEK* inhibitors	Total: 167,619 Drugs: 153,745	Total: 821,689 Drugs: 770,766	2.99	2.34	--	--	--	--	--	--
ICI- chemotherapy	Total: 268,802 Drugs: 255,909	Total: 1,220,967 Drugs: 1,150,107	4.97	3.71	51,134	201,778	73,561	290,279	−4899	−192,953

*BRAF*, v-Raf murine sarcoma viral oncogene homolog B; ICI, immune checkpoint inhibitor; INMB, incremental net monetary benefit; *MEK*, mitogen-activated protein kinase; QALY, quality-adjusted life-year; USD, US dollars.

**Table 3:** Exploratory analysis of *TP53* mutant and wild-type subgroups.

Strategy	Total Costs (USD)		QALYs	Incremental Cost per QALY (USD)		INMB (USD)	
	Taiwan	US		Taiwan	US	Taiwan	US
*TP53* mutant subgroup							
*BRAF* plus *MEK* inhibitors	141,885	692,547	1.95	--	--	--	--
ICI–chemotherapy	283,649	1,309,900	3.96	70,528	307,133	−960	−315,846
*TP53* wild-type subgroup							
ICI–chemotherapy	202,257	949,986	2.83	--	--	--	--
*BRAF* plus *MEK* inhibitors	242,914	1,261,544	3.28	90,924	696,767	−9356	−244,486

*BRAF*, v-Raf murine sarcoma viral oncogene homolog B; ICI, immune checkpoint inhibitor; INMB, incremental net monetary benefit; *MEK*, mitogen-activated protein kinase; QALY, quality-adjusted life-year; *TP53*, tumor protein p53; USD, US dollars.

## Data Availability

Study protocol and dataset: Not applicable. Statistical code: A partitioned survival model can be created from the information in the article. Codes are available on reasonable request to the corresponding author.

## Supplementary Materials

The following supporting information can be downloaded at https://www.mdpi.com/article/10.3390/curroncol33070384/s1, Figure S1: Model structure; Figure S2: Study and modeled progression-free survival and overall survival curves for patients under ICI-chemotherapy and *BRAF* plus *MEK* inhibitors; Table S1: The CHEERS 2022 checklist; Table S2: Doses and costs of drugs; Table S3: Parameter values for any grade AEs; Table S4: AIC and BIC for each parametric model of progression-free survival and overall survival; Table S5: Sensitivity analysis using alternative models to extrapolate the overall survival.
