# Supplementary material for: Cost-Effectiveness of First-Line Immunochemotherapy Versus BRAF Plus MEK Inhibitors in BRAFV600E-Mutated Metastatic Lung Cancer
Source: Curr Oncol. 2026 Jun 24;33(7):384. doi: 10.3390/curroncol33070384 (PMC13408117; doi:10.3390/curroncol33070384)
Supplement: Supplementary file 1 [file curroncol-33-00384-s001.zip › curroncol-4363749-supplementary.pdf]

Supplementary material

Cost-effectiveness of first-line immunochemotherapy versus *BRAF* plus *MEK* inhibitors in *BRAF*^{V600E}-mutated metastatic lung cancer

Figure S1. Model structure. *BRAF*, v-Raf murine sarcoma viral oncogene homolog B; ICI, immune checkpoint inhibitor; *MEK*, mitogen-activated protein kinase.

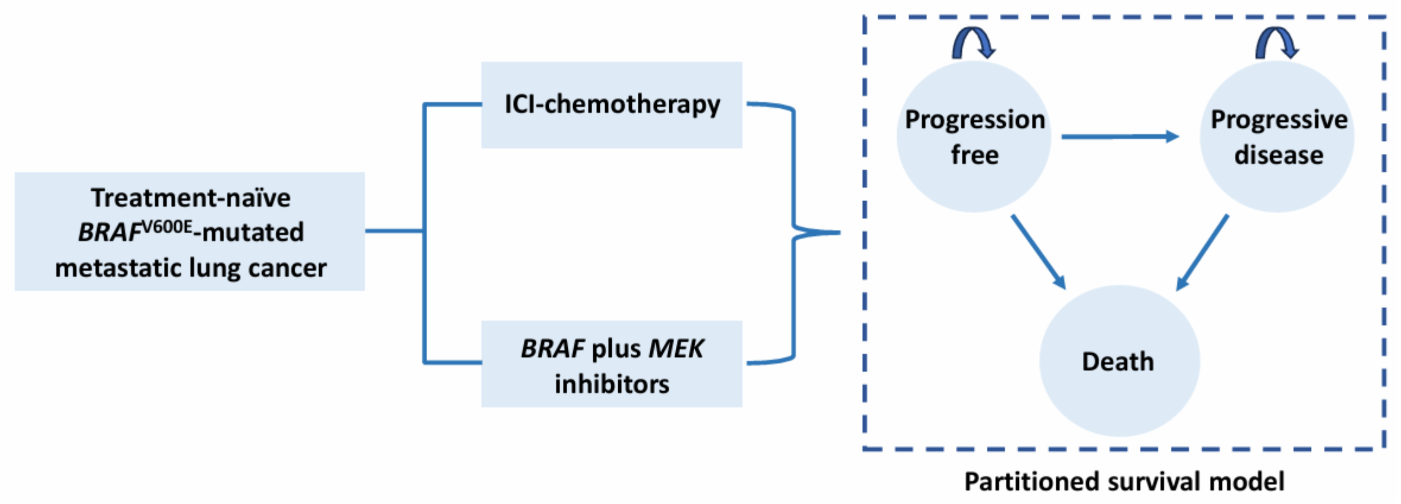

Figure S2. Study (blackened) and modeled (colored) progression-free survival and overall survival curves for patients under ICI-chemotherapy and *BRAF* plus *MEK* inhibitors. The dash lines represent the 95% confidence intervals of study survival curves. *BRAF*i, v-Raf murine sarcoma viral oncogene homolog B inhibitor; ICI-Chemo, immune checkpoint inhibitor plus chemotherapy; *MEK*i, mitogen-activated protein kinase inhibitor.

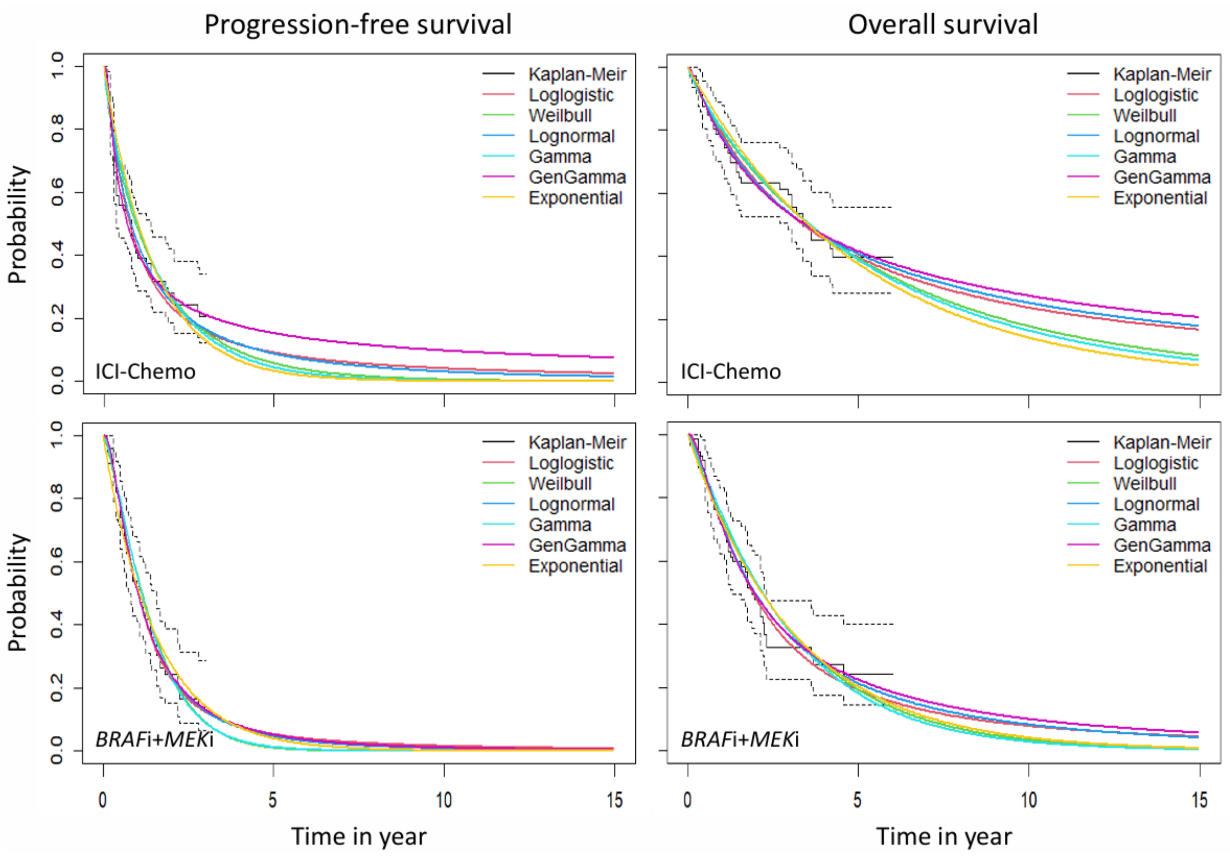

Table S1. The CHEERS 2022 checklist

Topic	No.	Item	Location where item is reported
**Title**			
	1	Identify the study as an economic evaluation and specify the interventions being compared.	Title
**Abstract**			
	2	Provide a structured summary that highlights context, key methods, results, and alternative analyses.	Abstract
**Introduction**			
**Background and objectives**	3	Give the context for the study, the study question, and its practical relevance for decision making in policy or practice.	Introduction
**Methods**			
**Health economic analysis plan**	4	Indicate whether a health economic analysis plan was developed and where available.	Materials and Methods, Model overview
**Study population**	5	Describe characteristics of the study population (such as age range, demographics, socioeconomic, or clinical characteristics).	Materials and Methods, Model overview
**Setting and location**	6	Provide relevant contextual information that may influence findings.	Materials and Methods, Cost and health utility
**Comparators**	7	Describe the interventions or strategies being compared and why chosen.	Materials and Methods, Model overview
**Perspective**	8	State the perspective(s) adopted by the study and why chosen.	Materials and Methods, Cost and health utility
**Time horizon**	9	State the time horizon for the study and why appropriate.	Materials and Methods, Model overview
**Discount rate**	10	Report the discount rate(s) and reason chosen.	Materials and Methods, Model overview
**Selection of outcomes**	11	Describe what outcomes were used as the measure(s) of benefit(s) and harm(s).	Materials and Methods, Base-case analysis
**Measurement of outcomes**	12	Describe how outcomes used to capture benefit(s) and harm(s) were measured.	Materials and Methods, Survival estimates & Cost and health utility
**Valuation of outcomes**	13	Describe the population and methods used to measure and value outcomes.	Materials and Methods, Survival estimates & Cost and health utility
**Measurement and valuation of resources and costs**	14	Describe how costs were valued.	Materials and Methods, Cost and health utility, 1st paragraph

Topic	No.	Item	Location where item is reported
**Currency, price date, and conversion**	15	Report the dates of the estimated resource quantities and unit costs, plus the currency and year of conversion.	Materials and Methods, Cost and health utility, 1st paragraph
**Rationale and description of model**	16	If modelling is used, describe in detail and why used. Report if the model is publicly available and where it can be accessed.	Materials and Methods, Model overview
**Analytics and assumptions**	17	Describe any methods for analysing or statistically transforming data, any extrapolation methods, and approaches for validating any model used.	Materials and Methods, Survival estimates
**Characterising heterogeneity**	18	Describe any methods used for estimating how the results of the study vary for subgroups.	Materials and Methods, Exploratory analysis of TP53 mutant and wild-type subgroups
**Characterising distributional effects**	19	Describe how impacts are distributed across different individuals or adjustments made to reflect priority populations.	Not applicable
**Characterising uncertainty**	20	Describe methods to characterise any sources of uncertainty in the analysis.	Materials and Methods, Deterministic and probabilistic analyses
**Approach to engagement with patients and others affected by the study**	21	Describe any approaches to engage patients or service recipients, the general public, communities, or stakeholders (such as clinicians or payers) in the design of the study.	Not applicable
**Results**			
**Study parameters**	22	Report all analytic inputs (such as values, ranges, references) including uncertainty or distributional assumptions.	Table 1 and Table S3
**Summary of main results**	23	Report the mean values for the main categories of costs and outcomes of interest and summarise them in the most appropriate overall measure.	Results, Base-case analysis, Exploratory analysis of TP53 mutant and wild-type subgroups & Tables 2 and 3
**Effect of uncertainty**	24	Describe how uncertainty about analytic judgments, inputs, or projections affect findings. Report the effect of choice of discount rate and time horizon, if applicable.	Results, Deterministic and probabilistic analyses & Figures 1 and 2 & Table S5
**Effect of engagement with patients and others affected by the study**	25	Report on any difference patient/service recipient, general public, community, or stakeholder involvement made to the approach or findings of the study	Not applicable
**Discussion**			
**Study findings, limitations, generalisability, and current knowledge**	26	Report key findings, limitations, ethical or equity considerations not captured, and how these could affect patients, policy, or practice.	Discussion
**Other relevant information**			

Topic	No.	Item	Location where item is reported
**Source of funding**	27	Describe how the study was funded and any role of the funder in the identification, design, conduct, and reporting of the analysis	Funding
**Conflicts of interest**	28	Report authors conflicts of interest according to journal or International Committee of Medical Journal Editors requirements.	Conflicts of interest

Table S2. Doses and costs of drugs

Drug	Dose	Unit price [a], USD		Cost per 3 weeks, USD	
		Taiwan	US	Taiwan	US
Carboplatin	490 or 545 mg (AUC: 5 mg/ml/min) every 3 weeks, up to four cycles	40	13	160	53
Pembrolizumab	200 mg every 3 weeks	1644	5769	3289	11,538
Pemetrexed	500 mg/m^2 * 1.68 or 1.90 m^2 every 3 weeks	250	49	500	99
Dabrafenib	150 mg twice daily	21	128	1781	10,763
Trametinib	2 mg once daily	85	556	1782	11,685
Encorafenib	450 mg once daily	28	133	3578	16,813
Binimetinib	45 mg twice daily	22	87	2734	10,998

[a] Carboplatin: 150 mg/vial; pembrolizumab: 100 mg/vial; pemetrexed: 500 mg/vial; dabrafenib: 75 mg/tablet; trametinib: 2 mg/tablet; encorafenib: 75 mg/tablet; binimetinib: 15 mg/tablet.
AUC, area under the concentration-time curve; USD, US dollars.

Table S3. Parameter values for any grade AEs

Parameter	Value	Range	Distribution	Source
Incidence of AE, ICI-chemotherapy				FRONT-BRAF study[1]
Pyrexia	6.8%	5.5–8.2%	beta	
Gastrointestinal	8.0%	6.4–9.5%	beta	
Skin	6.8%	5.5–8.2%	beta	
Fatigue	6.8%	5.5–8.2%	beta	
Liver	9.1%	7.3–10.9%	beta	
Rheumatologic	4.5%	3.6–4.5%	beta	
Pneumonitis	12.5%	10.0–15.0%	beta	
Renal	1.1%	0.9–1.4%	beta	
Endocrine	5.7%	4.5–6.8%	beta	
Incidence of AE, *BRAF* plus *MEK* inhibitors				FRONT-BRAF study[1]
Pyrexia	36.7%	29.4–44.1%	beta	
Gastrointestinal	24.5%	19.6–29.4%	beta	
Skin	19.4%	15.5–23.3%	beta	
Fatigue	10.2%	8.2–12.2%	beta	
Liver	9.2%	7.3–11.0%	beta	
Rheumatologic	9.2%	7.3–11.0%	beta	
Pneumonitis	6.1%	4.9–7.3%	beta	
Renal	2.0%	1.6–2.4%	beta	
Neurotoxicity	3.6%	2.9–4.3%	beta	
Endocrine	3.6%	2.9–4.3%	beta	
Pancreatic	1.5%	1.2–1.8%	beta	
Peripheral edema	1.5%	1.2–1.8%	beta	
Taiwan-Cost of AE management, USD				NHI claims analysis[2]
Pyrexia	5430	4344–6516	gamma	
Gastrointestinal	5794	4635–6953	gamma	
Skin	4233	3386–5080	gamma	
Fatigue	4113	3290–4936	gamma	
Liver	3682	2949–4418	gamma	
Rheumatologic	5248	4198–6298	gamma	
Pneumonitis	6891	5513–8269	gamma	
Renal	7839	6271–9407	gamma	
Neurotoxicity	9982	7986–11,978	gamma	
Endocrine	6217	4974–7460	gamma	
Pancreatic	5282	4226–6338	gamma	
Peripheral edema	5822	4658–6986	gamma	
US-Cost of AE management, USD				IQVIA PharMetrics® Plus claims analysis[3]
Pyrexia	2066	1653–2479	gamma	
Gastrointestinal	4351	3481–5221	gamma	
Skin	3168	2534–3802	gamma	
Fatigue	4260	3408–5112	gamma	
Liver	4477	3582–5372	gamma	
Rheumatologic	8324	6659–9989	gamma	
Pneumonitis	5753	4602–6904	gamma	
Renal	4260	3408–5112	gamma	
Neurotoxicity	6389	5111–7667	gamma	
Endocrine	5152	4122–6182	gamma	
Pancreatic	12,206	9765–14,647	gamma	

Peripheral edema	12,218	9774–14,662	gamma

AE, adverse event; *BRAF*, v-Raf murine sarcoma viral oncogene homolog B; ICI, immune checkpoint inhibitor; *MEK*, mitogen-activated protein kinase. NHI, National Health Insurance; USD, US dollars.

Table S4. AIC and BIC for each parametric model of progression-free survival and overall survival [a]

Parametric model	Progression-free survival		Overall survival	
	AIC	BIC	AIC	BIC
ICI-chemotherapy				
Loglogistic	134	138	191	196
Weibull	142	147	193	197
Lognormal	**131**	**134**	**190**	**194**
Gamma	144	148	193	198
Generalized gamma	127	135	191	198
Exponential	143	145	192	194
BRAF plus *MEK* inhibitors				
Loglogistic	135	140	**193**	**198**
Weibull	140	145	201	205
Lognormal	**134**	**139**	193	198
Gamma	138	143	200	205
Generalized gamma	136	143	195	202
Exponential	143	145	199	201

[a] Bold letters denote AIC and BIC for selected parametric models.
AIC, Akaike Information Criterion; BIC, Bayes Information Criterion; *BRAF*, v-Raf murine sarcoma viral oncogene homolog B; *MEK*, mitogen-activated protein kinase.

Table S5. Sensitivity analysis using alternative models to extrapolate the overall survival

ICI-chemotherapy vs *BRAF* plus *MEK* inhibitors	Incremental costs (USD)		Incremental QALYs	Incremental cost per QALY (USD)		INMB (USD)	
	Taiwan	US		Taiwan	US	Taiwan	US
Models for ICI-chemotherapy, loglogistic (base case) for *BRAF* plus *MEK* inhibitors							
Lognormal (base case)	101,184	399,278	1.38	73,561	290,279	-4899	-192,953
Weibull	81,723	287,274	1.06	77,041	270,815	-7469	-128,158
Loglogistic	96,329	371,338	1.30	74,271	286,308	-5540	-176,790
Exponential	72,471	234,018	0.91	79,539	256,844	-8691	-97,348
Models for *BRAF* plus *MEK* inhibitors, lognormal (base case) for ICI-chemotherapy							
Loglogistic (base case)	101,184	399,278	1.38	73,561	290,279	-4899	-192,953
Weibull	106,505	416,818	1.48	71,733	280,733	-2573	-194,106
Lognormal	97,251	386,318	1.29	75,111	298,367	-6617	-192,102
Exponential	104,846	411,350	1.45	72,273	283,555	-3298	-193,746

BRAF, v-Raf murine sarcoma viral oncogene homolog B; ICI, immune checkpoint inhibitor; INMB, incremental net monetary benefit; *MEK*, mitogen-activated protein kinase; QALY, quality-adjusted life-year; USD, US dollars.

References

1. Di Federico A, Wang K, Chen MF, et al. First-line immunotherapy with or without chemotherapy versus BRAF plus MEK inhibitors in BRAF(V600E)-mutated metastatic non-small-cell lung cancer (FRONT-BRAF): a multicentre, retrospective cohort study. *Lancet Oncol*. 2025;26(10):1357-1369. doi:10.1016/S1470-2045(25)00409-7
2. Lin CY, Wu TI, Yang SC. Estimating costs associated with adverse events in patients with advanced lung cancer. *Clinicoecon Outcomes Res*. 2024;16:761-769. doi:10.2147/CEOR.S489783
3. Patel A, Schuldt R, Sussell J. New estimates of the costs of adverse events in patients with cancer. *PLoS One*. 2025;20(9):e0332703. doi:10.1371/journal.pone.0332703
